# Characterization of the gut microbiota of invasive *Agrilus mali* Matsumara (Coleoptera: Buprestidae) using high-throughput sequencing: uncovering plant cell-wall degrading bacteria

**DOI:** 10.1038/s41598-019-41368-x

**Published:** 2019-03-20

**Authors:** Tohir A. Bozorov, Bakhtiyor A. Rasulov, Daoyuan Zhang

**Affiliations:** 1 Key Lab of Biogeography and Bioresource in Arid Land, Xinjiang Institute of Ecology and Geography, Chinese Academy of Sciences, 818 South Beijing Road, Urumqi, Xinjiang China; 2 Institute of Genetics and Plants Experimental Biology, Uzbek Academy of Sciences, Yukori-Yuz, 111226 Kibray, Tashkent Region Uzbekistan

## Abstract

The genus *Agrilus* comprises diverse exotic and agriculturally important wood-boring insects that have evolved efficient digestive systems. *Agrilus mali* Matsumara, an invasive insect, is causing extensive mortality to endangered wild apple trees in Tianshan. In this study, we present an in-depth characterization of the gut microbiota of *A. mali* based on high-throughput sequencing of the 16S rRNA gene and report the presence of lignocellulose-degrading bacteria. Thirty-nine operational taxonomic units (OTUs) were characterized from the larval gut. OTUs represented 6 phyla, 10 classes, 16 orders, 20 families, and 20 genera. The majority of bacterial OTUs belonged to the order Enterobacteriales which was the most abundant taxa in the larval gut. Cultivable bacteria revealed 9 OTUs that all belonged to Gammaproteobacteria. Subsequently, we examined the breakdown of plant cell-wall compounds by bacterial isolates. Among the isolates, the highest efficiency was observed in *Pantoea* sp., which was able to synthesize four out of the six enzymes (cellulase, cellobiase, β-xylanase, and β-gluconase) responsible for plant-cell wall degradation. One isolate identified as *Pseudomonas orientalis* exhibited lignin peroxidase activity. Our study provides the first characterization of the gut microbial diversity of *A. mali* larvae and shows that some cultivable bacteria play a significant role in the digestive tracts of larvae by providing nutritional needs.

## Introduction

Insecta is the largest class among the invertebrates, and species have the ability to feed on different food sources through specialized digestive tracts. The beetles (Coleoptera), with approximately 400,000 species, are the largest order^1^. The family Buprestidae, also known as the jewel beetle family, has approximately 3000 species, and many of them are invasive exotic insects^2,3^. Many Buprestidae damage trees, leading to mortality, and are thus considered agriculturally significant pests^2^. Often, the larvae of Buprestidae parasitize the woody portion of plant tissue, especially the phloem (rarely the xylem), via digestion of plant cell-wall polymers (lignocellulosics). These polymers represent one of the most abundant renewable resources on the planet^4,5^.

Insects have symbiotic associations with diverse and complex microorganisms, including resident and transient bacteria, fungi, actinomycetes, and archaea^6^. Over the past decades, there has been an increasing number of works on the gut microbiota of wood-boring beetles because they play a key role in insect physiology and adaptation to the environment and their ecological relevance^7^. The gut microbiota-host relationship ranges among symbiotic interactions, i.e., from parasitism to mutualism^8,9^. Microbial symbionts contribute or take part in various physiological processes in insects, including growth, nutrition and vitamin production, development, pathogenesis, immunity, production of components of pheromones, and adaptability to the environment^8–10^. The gut microbial community is known to be diverse and differs with insect species, different stages and periods of the host life cycle^9,11^.

In wood-boring beetles, the gut microbiota is prominent in the digestive tract and plays essential roles in compensating for dietary deficiencies and compound detoxification^12–14^. Moreover, many wood-boring beetles have become significant forest pests that cause extensive mortality of economically important trees. Therefore, exploration of their feeding capabilities is essential for developing pest management programs^15–17^. The digestive tract and gut microbiota are mainly based on the food source and tissue type. Moreover, certain microbial communities might adapt to the endointestinal lifestyle and have developed mutualistic relationships for host survival. However, little is known about wood-boring larvae feeding behaviour, digestive tracts, gut microbiota, diversity and the symbiotic interactions with insects that develop within the stem phloem and cambium tissues. Furthermore, the potential role of gut microorganisms in lignocellulosic digestion by wood-boring larvae has been thoroughly explored. Therefore, larvae harbour diverse microbial communities, and exploring the role of the gut microbiota helps in understanding insect digestion of plant cell-wall compounds. A symbiotic interaction between gut microbiota and insects likely aids in the digestion of plant cell-wall polymers and provides nutritional supplements for the hosts.

An invasive wood-boring beetle, *Agrilus mali* Matsumara (Coleoptera: Buprestidae), which is believed to have been introduced from East China in the early 1990s, has caused extensive mortality of wild apple in Tianshan (West China) forests, resulting in severe environmental losses^18–20^. Beetles of *A. mali* lay eggs on or in bark crevices of apple trees, and after hatching, neonates immediately bore into the bark and start feeding on the phloem and cambium. The larval stage is the most destructive for the tree because larvae form serpentine galleries throughout the phloem, resulting in disruption of nutrient movement and causing death^18–20^. The purpose of this study was to characterize the gut microbial communities in depth and to explore the lignocellulolytic activity of cultivable bacteria from fourth-fifth instar *A. mali* larvae. Characterization of the microbial communities was based on culture-dependent and culture-independent approaches. Six lignocellulolytic assays were used to test cultivable gut bacteria for their ability to degrade plant cell-wall polymers. We found that the *A. mali* larvae gut includes diverse bacterial species belonging to six phyla, and the most abundant among Proteobacteria species were able to break down plant cell-wall components.

## Results

### Identification and phylogenetic diversity of bacteria associated with the *A. mali* larvae gut

The gut microbiota were extracted from the guts of ten *A. mali* larval insect specimens. High-throughput sequencing of bacterial DNAs of gut microbiota resulted in a total of 96, 206 ± 1319.5 raw reads. After data quality filter processing, the number of quality-controlled reads was 90940 ± 2104.5. The average sequence length of the amplicon was 428 nucleotides. The total number of operational taxonomic units (OTUs) assigned during analysis was 39 bacterial OTUs using 98% similarity in culture-independent methods (Table 1). Analysis revealed that 37 OTUs were found in the gut and 2 OTUs (30 and 35) were detected on the entire sterilized larval body surface.

**Table 1:** Bacterial taxa from guts of *A. mali* larvae identified by 16S rRNA high-throughput sequencing analysis.

OTU	Phylum	Order	Family	Genus	Predicted species (GenBank #)	Abundance
38	Acidobacteria				JF986535*	2
30					GU015920*	3
9	Actinobacteria	Propionibacteriales	Propionibacteriaceae	*Propionibacterium*	*P. acnes* CP023676; *R. erythropolis* MH298510*	26
19		Actinomycetales	Nocardiaceae	*Rhodococcus*	*R. qingshengii* MH064222*; *R. degradans* KY992558*	12
21	Bacteroidetes	Bacteroidales	Prevotellaceae	*Prevotella*	*Revotella* sp. MG801743	14
14		Bacteroidales	Rikenellaceae	*Rikenella* sp.	*Rikenella* sp. KC417282***	39
26		Bacteroidales	Porphyromonoadaceae	*Odoribacter*	*Odoribacter* MG052424*	3
11	Firmicutes	Lactobacillales	Lactobacillaceae	*Lactobacillus*	*L. johnsonii* MH393024;	23
20		Lactobacillales	Lactobacillaceae	*Lactobacillus*	*L. intestinalis* LC096206*	12
23		Clostridiales			MG802288	10
4		Clostridiales			AB751303	156
31		Clostridiales	Lachnospiraceae		FJ833589*	8
37		Oscillospirales	Ruminococcaceae		AB700360*	7
15		Oscillospirales	Ruminococcaceae		KY664658*	19
25		Oscillospirales	Ruminococcaceae	*Negativibacillus*	*N. massiliensis* NR_147378**	2
39	Proteobacteria	Burkholderiales	Sutterellaceae	*Sutterella*	*Sutterella* sp. KX833879	8
13		Desulfovibrionales	Desulfovibrionaceae	*Bilophila*	*Bilophila* sp. KP055112	6
12		Desulfovibrionales	Desulfovibrionaceae	*Desulfovibrio*	*Desulfovibrio* sp. KF760539*	47
3		Campylobacterales	Campylobacteraceae	*Arcobacter*	*Arcobacter* sp. FN397894	466
10		Campylobacterales	Helicobacteraceae	*Helicobacter*	*H. typhlonius* NR_041748; *H. apri* KP120975*; *H. mastomyrinus* NR_115314*; *H. japonicas* NR_149210*	12
5		Pseudomonadales	Moraxellaceae	*Acinetobacter*	*A. nosocomialis* CP029351; *A. pittii* KY941128; *A. calcoaceticus* MF149066	113
24		Enterobacteriales	Enterobacteriaceae	*Erwinia*	*Erwinia* sp. MF525796*	254
22		Enterobacteriales	Enterobacteriaceae	*Erwinia*	*E. tasmaniensis* KF574916*	16
17		Enterobacteriales	Enterobacteriaceae	*Escherichia*	*E. coli* CP008805	7
1		Enterobacteriales	Enterobacteriaceae	*Pantoea*	*P. agglomerans* MH158658	77651
18		Enterobacteriales	Enterobacteriaceae	*Pantoea*	*P. agglomerans* KT075213*	35003
8		Enterobacteriales	Enterobacteriaceae	*Pantoea*	*P. vagans* MH211327*; *P. agglomerans* MH190052	24347
32		Enterobacteriales	Enterobacteriaceae	*Pantoea*	*P. agglomerans* MH165381*; *P. vagans* MG819435*	1646
2		Pseudomonadales	Pseudomonadaceae	*Pseudomonas*	*Ps. congelans* LT547855; *Ps. syringae* KR922152; *Ps. cannabina* JN167950; *Ps. viridiflava* HE588020	2
7		Pseudomonadales	Pseudomonadaceae	*Pseudomonas*	*Ps. gessardii* MH398505; *Ps. reactans* MH396741; *Ps. synxantha* CP011117; *Ps. brenneri* MF509842; *Ps. azotoformans* KY939740; *Ps. libanensis* KY939752; *Ps. fluorescens* MG977684; *Ps. paralactis* MG952589	101
33		Pseudomonadales	Pseudomonadaceae	*Stenotrophomonas*	*S. pavanii* MF375923; *S. maltophilia* MH396764	2
6		Vibrionales	Vibrionaceae	*Vibrio*	*V. scophthalmi* MG456764*; *V. ichthyoenteri* KJ817452*	108
28		Vibrionales	Vibrionaceae	*Vibrio*	*V. azureus* CP018617*; *V. ichthyoenteri* NR_117888*; *V. comitans* KT023539*; *V. furnissii* KR270195*; *V. ponticus* KF193915	13
29	Spirochaetes	Spirochaetales	Spirochaetaceae	*Spirochaeta*	*Spirochaeta* sp. DQ340184**	12
27		Spirochaetales	Spirochaetaceae		*Spirochaeta* sp. DQ340184***	23
36	Unknown				Uncultured bacterium MF259861	5
35	Unknown				Uncultured bacterium MF081102	4
34	Unknown				Uncultured bacterium MF260097	

*Similarity between 97% < 100%; **similarity between 95% < 97%; less than 95% similarity.

**Figure 1:**
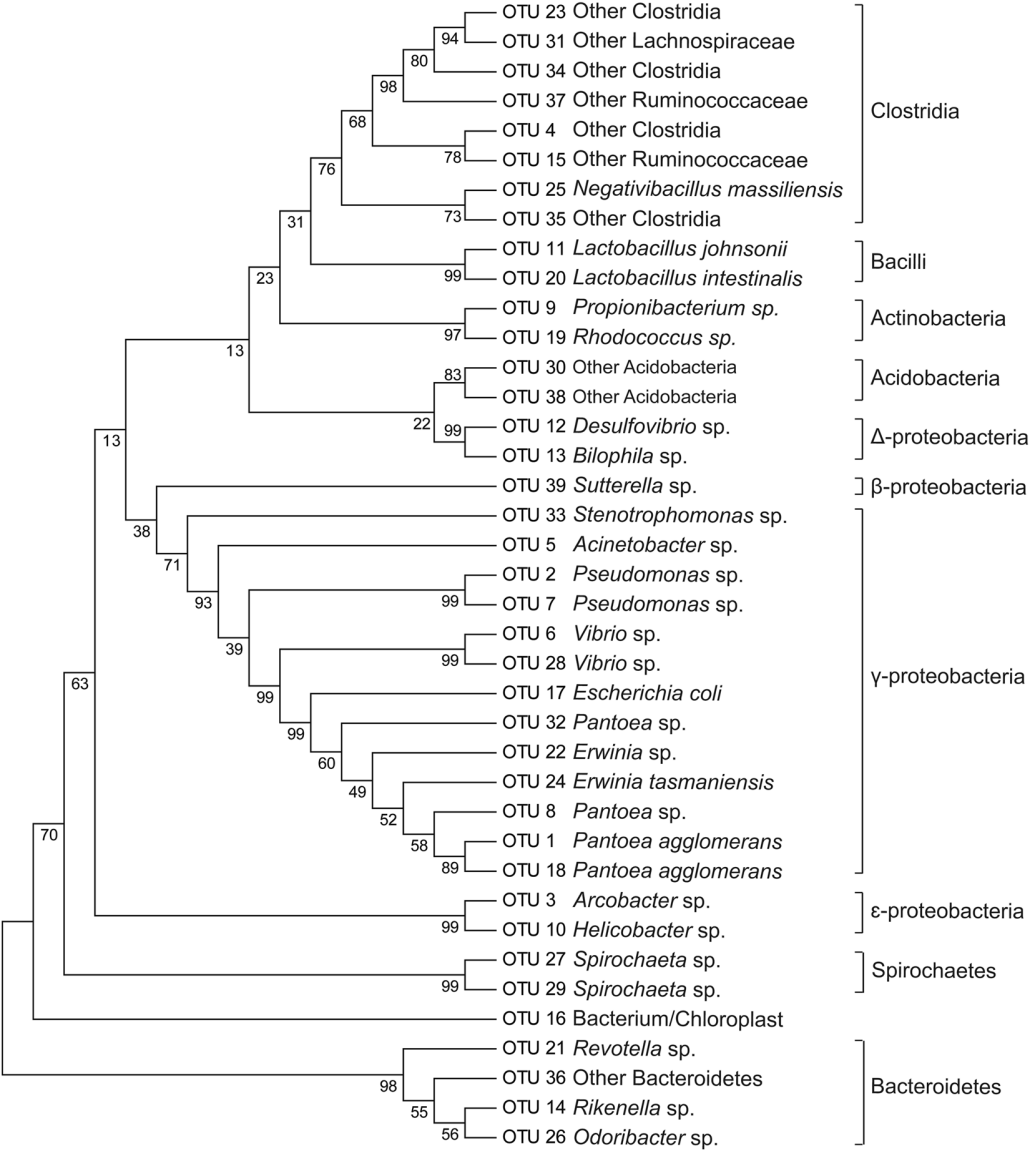
Neighbour-joining tree of partial 16S rRNA sequences retrieved from bacterial community of larvae of *A. mali* using a culture-independent approach. The evolutionary history was inferred using the UPGMA method. The percentage of replicate trees in which the associated taxa clustered together in the bootstrap test (1000 replicates) are shown next to the branches. The evolutionary distances were computed using the Tajima-Nei method and are in units of the number of base substitutions per site. Evolutionary analyses were conducted in MEGA7.

**Figure 2:**
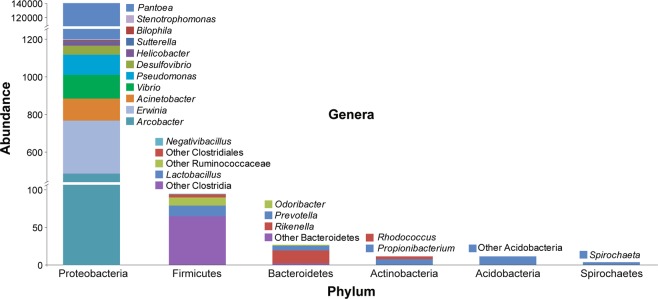
Relative OTU abundance in the *A. mali* larval gut. The abundance refers to the relative proportion of OTUs containing genera within the distribution of each parent phylum displayed on the x-axis.

The identification of OTUs was performed by comparison with publicly available sequences in GenBank via BLASTN algorithm search. BLAST analysis revealed that most of the OTUs were distributed into six phyla: Acidobacteria, Actinobacteria, Bacteroidetes, Firmicutes, Proteobacteria, and Spirochaetae; ten classes: Acidobacteria, Actinobacteria, Bacteroidetes, Bacilli, Clostridia, Betaproteobacteria, Deltaproteobacteria, Epsilonproteobacteria, Gammaproteobacteria, Spirochaetes; sixteen orders: Acidobacteriales, Propionibacteriales, Actinomycetales, Bacteroidales, Lactobacillales, Burkholderiales, Oscillospirales, Clostridiales, Burkholderiales, Campylobacterales, Pseudomonadales, Enterobacteriales, Pseudomonadales, Xanthomonadales, Vibrionales, and Spirochaetales; twenty families: Acidobacteriaceae, Propionibacteriaceae, Nocardiaceae, Prevotellaceae, Rikenellaceae, Porphyromonadaceae, Lactobacillaceae, other Clostridiales, Lachnospiraceae, Ruminococcaceae, Sutterellaceae, Desulfovibrionaceae, Campylobacteraceae, Helicobacteraceae, Moraxellaceae, Enterobacteriaceae, Pseudomonadaceae, Xanthomonadaceae, Vibrionaceae, and Spirochaetaceae; and twenty genera: other Acidobacteria, *Propionibacterium, Rhodococcus, Prevotella, Rikenella, Odoribacter, Lactobacillus*, other Clostridiales, other Lachnospiraceae, other Ruminococcaceae, *Negativibacillus, Sutterella, Bilophila, Desulfovibrio, Arcobacter, Helicobacter, Acinetobacter, Erwinia, Escherichia, Pantoea, Pseudomonas, Stenotrophomonas, Vibrio*, and *Spirochaeta*. Phylogenetic analysis of the sequenced bacterial species demonstrated that the two largest clades were Proteobacteria and Firmicutes followed by Acidobacteria, Actinobacteria, Bacteroidetes and Spirochaetes (Fig. 1). The Gammaproteobacteria dominated the larval gut libraries (99.3%) and within the class, the most abundant genus was *Pantoea* (98.8%) (Fig. 2). Within the Firmicutes, the class Clostridia was the most abundant. Bacterial OTUs also contained sequences similar to less-characterized phyla. Two OTUs with low abundance demonstrated close similarity with Acidobacteria, two with Spirochaetes, and one with Bacteroidetes species (Table 1). Four OTUs were highly similar to *Pantoea* sp. (98.8%). Other genera included *Arcobacter* (0.33%), two isolates of *Erwinia* (0.19%), two isolates of *Clostridium* (0.11%), *Lachnospiraceae* (0.11%), two isolates of *Vibrio* (0.09%), two isolates of *Pseudomonas* (0.07%), *Acinetobacter* (0.02%), two isolates for *Lactobacillus* (0.02%), *Rikenella* (0.02%), *Spirochaeta* (0.02%), *Propionibacterium* (0.02%), and other genera with abundances of less than 0.01%. The amount of unidentified bacteria was 0.11%.

### Diversity of cultivable bacteria from the *A. mali* larvae gut

**Figure 3:**
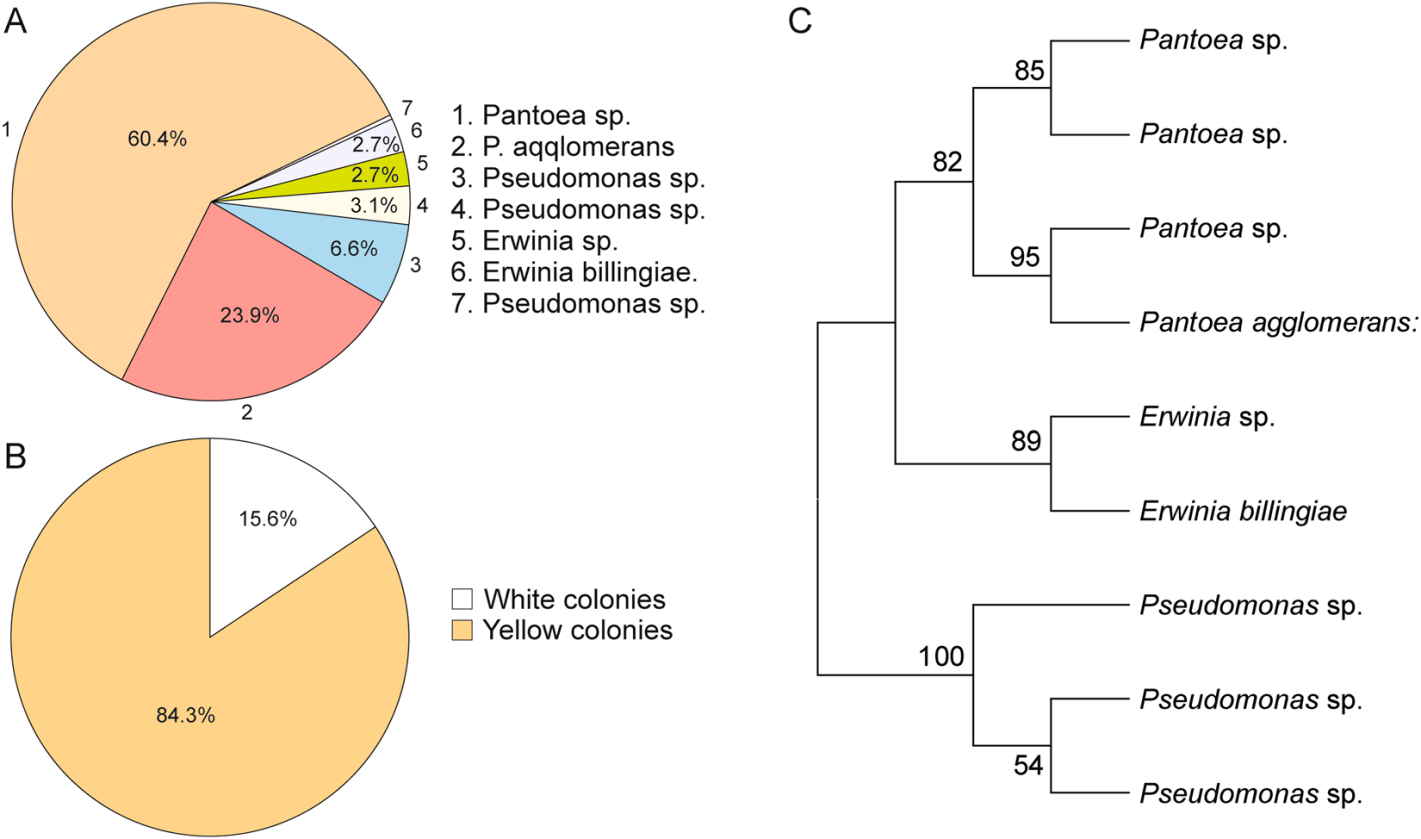
Diversity, abundance and clustering analysis of cultivable gut bacteria. Distribution of the cultivable bacterial community of the gut of *A. mali* larvae by their abundance (**A**). Coloration of cultivable bacterial isolates (**B**). Clustering analysis of sequenced cultivable bacteria conducted in MEGA7 (**C**).

A total of 288 screened bacterial colonies from *A. mali* larvae were studied. Selection of a colony was randomly based on the morphotype of the colonies. Individual DNA was extracted from each colony, and 16S rRNA sequencing of DNA was performed by traditional Sanger sequencing methods. Sequence analysis of the 16S rRNA gene showed 9 OTUs, identified as various *Pantoea, Erwinia* and *Pseudomonas* species, the details of which are shown in Table 2. The identification of sequences by BLAST searches of cultivable bacteria revealed that among the 288 isolates, the most abundant genus was *Pantoea* (84.3%). *Pseudomonas* and *Erwinia* represented 9.7% and 5.5% of the cultivable bacteria, respectively (Fig. 3A). All cultivable bacteria belonged to the class Gammaproteobacteria but showed diversity in colony types (Fig. 3B). A subsequent morphological analysis revealed that 84% of all colonies displayed yellow coloration that corresponded to *Pantoea* sp. Colonies of *Pantoea* sp. were smooth, translucent, and convex with entire margins on NA plates with non-pigmented or yellow colonies. Two isolates of *Erwinia* sp. showed different colony types. Generally, both were circular, smooth and white but different in colony margin with one being entire and the other lobate. One produced a weakly diffusible pink pigment. *Pseudomonas* sp. showed white colony colour as well either circular or irregular forms.

**Table 2:** Bacteria isolated from guts of *A. mali* larvae identified by 16S rRNA sequence analysis.

Number of isolates	Genus	Predicted species	GenBank accession	Similarity (%)
8	*Erwinia*	*E. billingiae*	KJ004476	100
8	*Erwinia*	*E. persicina*	MF193907	100
		*E. rhapontici*	KF500098	100
1	*Pseudomonas*	*Ps. syringae*	MG720019	100
		*Ps. amygdale*	CP020351	100
		*Ps. cerasi*	LT963395	100
		*Ps. congelans*	JQ320090	100
		*Ps. savastanoi*	DQ318862	100
19	*Pseudomonas*	*Ps. orientalis*	CP018049	100
8	*Pseudomonas*	*Ps. fluorescens*	KT215480	99
		*Ps. synxantha*	CP011117	99
		*Ps. libanensis*	LT629699	99
		*Ps. gessardii*	MF077145	99
		*Ps. azotoformans*	LT629702	99
		*Ps. chlororaphis*	CP011020	99
174	*Pantoea*	*P. agglomerans*	MH190052	99
		*P. vagans*	KC139414	99
		*P. herbicola*	U80202	99
		*P. ananatis*	KC178592	99
		*P. conspicua*	HF562884	99
		*P. brenneri*	KX588583	99
66	*Pantoea*	*P. agglomerans*	MH158730	99
3	*Pantoea*	*P. agglomerans*	JX077098	99
		*P. vagans*	KP099965	99
		*P. ananatis*	KR361756	99
3	*Pantoea*	*P. agglomerans*	MH158730	99
		*P. vagans*	CP014129	99

Sequence and clustering analyses demonstrated that the *Pantoea* genus included four species (Fig. 3C). The most abundant *Pantoea* species showed close similarity to *P. agglomerans, P. vagans, P. herbicola, P. ananatis, P. conspicua*, and *P. brenneri* (60.4%). Other groups showed similarity to *P. agglomerans* (22.9%); *P. agglomerans, P. vagans*, and *P. ananatis* (1%) as well as *P. agglomerans* and *P. vagans* (1%). The *Pseudomonas* genus clustered into three groups. Sequence analysis of the first showed high sequence similarity to *Ps. syringae, Ps. amygdale, Ps. cerasi, Ps. congelans* and *Ps. savastanoi* (0.34%); the second group to *Ps. orientalis* (6.59%); and the third group to *Ps. fluorescens, Ps. synxantha, Ps. libanensis, Ps. gessardii, Ps. azotoformans* and *Ps. chlororaphis* (2.77%). Clustering of *Erwinia* species showed two groups that were highly similar to *E. billingiae* (2.77%) and to *E. persicina* and *E. rhapontici* (2.77%) (Table 2).

### Lignocellulolytic activity

**Figure 4:**
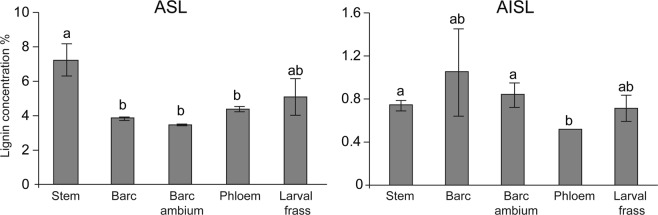
Lignin content in different apple tissues and larval frass. ASL, amino acid soluble lignin; AISL, amino acid insoluble lignin. Values shown are the mean (±SE) of 100 replicates. Different letters show significant differences among months of each stage as determined by one-way ANOVA, followed by a Fisher PLSD post hoc test (P ≤ 0.05).

Furthermore, we tested 288 cultivable microbial isolates for their ability to break down plant cell components. Nearly 90% of the bacterial isolates showed lignocellulolytic activity, and cellulose degradation was the most common activity observed among the isolates. In total, 260 isolates showed cellulolytic activity, whereas 242 isolates exhibited xylanase activity, 251 isolates exhibited glucanase activity, 242 isolates exhibited cellobiase activity, and 19 isolates exhibited lignin peroxidase activity (Table 3). The isolates did not show laccase activity. In this context, *Pantoea* spp. showed higher activity for degrading plant cell wall components than other Proteobacteria. In particular, these species were able to synthesize cellulase, cellobiase, β-xylanase, and β-gluconase enzymes. On the other hand, both identified isolates of *Erwinia*, namely, *Erwinia* sp. and *E. billingiae* actively degraded carboxymethylcellulose. It should be noted that, apart from carboxymethylcellulose degradation ability, *E. billingiae* also produced glucanase. Representatives of *Pantoea*, *Erwinia* and *Pseudomonas* were not able to degrade Remazol Brilliant Blue molecules except from *Pseudomonas orientalis*. They also did not demonstrate laccase activity in the ABTS test (Table 3). However, the lignin levels in different apple tree tissues as well as larval frass showed different abundances (Fig. 4). The results demonstrated that ASL accumulated highly in the stem, but AISL did not differ among the tissues. In both AISL and ASL, there was less phloem.

**Table 3:** Distribution of enzymatic activities of gut microbial isolates obtained from larvae of *A. mali*.

Genus/Species	Colony	CMC^a^	Xylanase	Glucanase	Cellobiase	RBBR^b^	LAC^c^
*Pantoea* sp.^d^	Yellow	171	169	171	171	0	0
*Pantoea* sp.	Yellow	3	3	3	3	0	0
*Pantoea* sp.	Yellow	3	3	3	3	0	0
*P. aqqlomerans* ^e^	Yellow	66	66	66	66	0	0
*Erwinia* sp.	White	6^f^	1	8	0	0	0
*E. billingiae*	White	8^g^	1	1	0	0	0
*Pseudomonas* sp.	White	0	0	0	0	0	0
*Ps. orientalis*	White	0	0	0	0	19	0
*Pseudomonas sp*.	White	3^h^	2	0	0	0	0

^a^Cellulose activity on CMC agar.

^b^Lignin peroxidase activity in MEA-RBBR.

^c^Laccase activity on ABTS test.

^d^80 isolates has less cellulotic, 6 – xylanase, and 8-glucanase activities.

^e^49 isolates has less cellulotic, 3 – xylanase, and 2-glucanase activities.

^f^3 isolates has less cellulotic acivity.

^g^2 isolates has less cellulotic acivity.

^h^2 isolates has less cellulotic acivity.

## Discussion

In this study, we explored the diversity of the microbial community that colonizes the larval gut of the invasive wood-borer *A. mali* to elucidate the digestive process of larvae for plant cell-wall component breakdown. We isolated gut microorganisms using culture-dependent and culture-independent approaches for high-throughput sequencing analysis to determine microbial diversity and to evaluate their lignocellulosic degradation ability. However, the main limitation of this study was the artificial media, which facilitated growth of only a small number of bacterial species present in the larval gut. Phylogenetic analysis demonstrated that the *A. mali* gut microecosystem is highly diverse with various species abundances.

Overall, our results demonstrated that the gut bacterial community of *A. mali* larvae is relatively complex. To identify the gut microbiota of *A. mali* larvae, the variable regions of the 16S rRNA gene were analysed by high-throughput sequencing, and the use of variable regions has already been shown in several studies^21–23^. In total, we sequenced gut bacterial associates, obtaining 90940 clean reads from culture-independent methods and 288 microorganisms from culture-dependent approaches. We observed a great diversity of bacterial communities representing thirty-nine OTUs belonging to five phyla. Two OTUs were detected in the whole body and 37 OTUs in the larval gut. Two OTUs could be found in the mouth or other parts of the larvae. Approximately 99% of the species found in the larvae gut were β-, ɛ-, Δ-, and γ-proteobacteria and Clostridia. These data are consistent with other reported studies of wood boring beetles^17,24,25^. In comparison with other wood-boring species whose gut bacterial community was studied^17,21,26–29^, *A. mali* larvae had a complex microbiota; however, compared to other wood-boring beetles of Scarabaeidae, Passalidae, Elateridae, Cerambycidae, and Tenebrionidae, the gut microbial diversity of *A. mali* seems richer in bacterial species^27,30^. Apparently, the gut diversity of *A. mali* differed from that reported for another *Agrilus* species, *A. planipennis*^17^. Interestingly, these *Agrilus* species harbour different bacterial species that belong to almost the same phyla. For example, species belonging to Acidobacteria, Actinobacteria, Firmicutes and Bacteroidetes differed from each other. However, *A. mali* and *A. planipennis* commonly share *Pseudomonas, Erwinia* and *Pantoea* species.

The bacterial community of *A. mali* was mainly dominated by Proteobacteria, accounting for 99.7%, with other bacterial classes Firmicutes, Acidobacteria, Actinobacteria, and Bacteroidetes, accounting for less than 1%. To the best of our knowledge, this type of bacterial diversity and large differences in bacterial abundance are reported here for the first time. For example, closely related emerald ash borer *A. planipennis* larvae harboured 44% Proteobacteria and 38% Firmicutes^27^. Our study demonstrated that Firmicutes were the second most predominant phylum after Proteobacteria in the *A. mali* larval gut. In general, our study validates other studies that reported a predominance of Proteobacteria and Firmicutes in wood-boring beetles^12,17,21,27^, but our results differed in regard to species abundance. Furthermore, recent work by Zhang *et al*.^31^ reported bacterial communities of *A. mali* larvae fed on leaves of different apple (*Malus*) species under laboratory conditions^31^. The authors demonstrated that species of γ-proteobacteria accumulated more when larvae were fed *M. halliana* leaves compared to *M. pumila* leaves.

High bacterial diversity in the gut of *A. mali* larvae could indicate that larvae feed on the nutrient-rich cambium and phloem. Colman *et al*. provided evidence that the diet of the host can affect an organism’s gut microbial community^11^. This is evidenced by the low gut microbial diversity of the red palm weevil *Rhynchophorus ferrugineus* Olivier (Coleoptera: Curculionidae), which feeds on nutrient-poor palm tissues and sap that contains mainly sucrose and glucose^32^. Sugars affect the complexity of the gut microbiota^33^, which may account for the low gut bacterial diversity of field sampled larvae^21^. Conversely, complex substrates such as plant cell-wall lignocellulosics account for the complex bacterial community of the gut^11^.

Gammaproteobacteria were the most abundant among the identified gut microbial taxa. However, *Pantoea* had highly dominant species among all identified bacteria (99%). There were four *Pantoea* sp. identified in the gut, and two of them were identified as two different strains of *P. agglomerans*. These data were consistent with the sequencing data of cultivable bacteria that also showed four *Pantoea* species. The results revealed that all four *Pantoea* species had the ability to break down plant cell-wall polymers. However, this genus did not show lignin-degrading ability. This could be explained by the fact that phloem tissue contains less lignin than xylem tissue. Lourenço *et al*. reported that lignin composition and structure differed between xylem and phloem^34^. Our results also indicated that both AISL and ASL levels were significantly lower in phloem and cambium compared to other tissues. Larvae cannot digest the hard lignified xylem. Among the bacterial species, only *Ps. orientalis* showed the capacity for lignin peroxidase activity, which may help release cellulosics from lignin in the phloem. Interestingly, the lignin content was relatively higher in larval frass compared to the phloem, cambium and bark tissues. This indicates that the frass sample contains less cellulosics due to degradation by the gut bacteria and leads to an increased level of lignin.

The diversity and abundance of the gut bacterial community relies on substrates and their competition with each other. For example, along with *Pantoea*, species of *Erwinia* (levels of which were estimated at 0.19%) are also capable of degrading plant cell-wall components. On the other hand, bacterial species could secrete antibacterial compounds when competing for the same substrate. It has been reported that *P. agglomerans* possesses unique metabolic capabilities to produce antibiotics^35–37^. These antibiotics could be used for combating fungi and bacterial species present in the gut. Apparently, next-generation sequencing analysis did not detect any fungi in the *A. mali* gut. The antibiotic herbicolin I, produced by *P. agglomerans* and *P. vagans*, has been reported to suppress the pathogen *E. amylovora*, a pathogen of apple and pear species^38^.

In conclusion, the results of the current study provide new insight into the diversity of microbial communities and their role in plant cell-wall biopolymer breakdown. This helps to highlight the mechanism of digestion of plant compounds in the larval gut. This work demonstrates that the microbial community of larvae is complex and mainly dominated by γ-proteobacteria. Within the γ-proteobacteria, the *Pantoea* are the most dominant species in the gut that likely engage in insect-bacteria symbiosis. Moreover, the gut bacterial community might participate in early invasive abilities, leading to host survival in new regions. However, to better understand invasion histories, *A. mali* samples collected from natural habitats (Eastern China) and invaded regions (Western China) should be compared in regard to gut microbial diversity. Furthermore, the knowledge gained from these studies could be exploited by describing the enzymatic capabilities of gut microorganisms and their roles in host ecophysiology, developing pest management by seeking antagonistic Enterobacteria to inhibit cellulose-degrading bacteria and developing enzyme systems for biotechnological applications.

## Materials and Methods

### Insect collection and dissection

*Agrilus mali* insect specimens were collected from April to May 2017 in a wild apple nursery in Mohe Village (43°51N, 82°15W), Gongliu County, Ili-Kazakh District, Xinjiang-Uyghur Autonomous Province, China PR. The first record of *A. mali* in western China was reported by Ji *et al*.^18^; *A. mali* was later described by DNA barcoding in our recently accepted work^20^. Insect larvae were collected randomly from infested trees. All larvae were placed inside vented polyethylene containers with woody material (apple twigs) and transported to the laboratory. Insects were surface sterilized in 95% ethanol for 5 s to remove surface microbes^39^. Larvae were washed five times with sterile Milli-Q water. Surface-sterilized larvae were dissected under aseptic conditions using a scalpel and forceps to extract the digestive tract in 10 mM phosphate-buffered saline (PBS-buffer) under a sterile laminar flow hood^17^. The larval head and last segment were severed, and the gut was transferred into a 1.5 mL tube with 100 µL PBS buffer (NaCl - 0.137 M, KCl - 0.0027 M, Na_2_HPO_4_ - 0.01 M; KH_2_PO_4_ – 0.0018 M; pH 7.4) and homogenized using a sterile plastic pestle. Surface sterilized whole larvae were homogenized under aseptic conditions. Homogenates were stored at −20 °C for further culture-independent methods.

### Sequencing of non-cultured bacteria

A 16S rRNA gene library from the larvae gut was constructed using ten pooled homogenized guts/whole larvae. Bacterial DNA isolated from the pooled samples was homogenized using the CTAB/SDS method. DNA concentration and purity were monitored on 1% agarose gels. According to the concentration, DNA was diluted to 1 ng/μL using sterile water. Extracted genomic DNA was sent to Novogene China (www.novogene.com) for Illumina generation sequencing. Briefly, amplicons for the 16S rRNA/18SrRNA/ITS genes of distinct regions were amplified using specific primer pairs as follows: for the 16S V4 region, 515F/806R (5′-GTGCCAGCMGCCGCGGTAA-3/5′-GGACTACHVGGGTWTCTAAT-3); for 18S V4 - 528F/706R (5′-GCGGTAATTCCAGCTCCAA-3′/5′-AATCCRAGAATTTCACCTCT-3′); for 18S V9- 1380F/1510R (5′-CCCTGCCHTTTGTACACAC-3′/5′-CCTTCYGCAGGTTCACCTAC-3′) with the barcode. All PCRs were carried out with Phusion® High-Fidelity PCR Master Mix (New England Biolabs). PCRs were carried out in a volume of 30 μL with 15 μL of Master Mix, 0.2 μM of forward and reverse primers, and approximately 10 ng of template DNA. PCR conditions included an initial denaturation for 1 min at 98 °C, 30 cycles of 98 °C for 10 s (denaturation), 50 °C for 30 s (annealing), and at 72 °C for 1 min (elongation), and a final extension at 72 °C for 5 min.

Quantification and quality assessment of PCR products were carried out in the same volume of 1X loading buffer containing SYBR green with PCR products on a 2% agarose electrophoresis gel (Sigma-Aldrich, USA) for visualization. Samples with bright distinct strips between 400–450 bp were chosen for further experiments. Next, PCR products were purified with a Qiagen Gel Extraction Kit (Qiagen, Germany).

Sequencing libraries were generated using the TruSeq® DNA PCR-Free Sample Preparation Kit (Illumina, USA) following the manufacturer’s recommendations, and index codes were added. The library quality was assessed on the Qubit@ 2.0 Fluorometer (Thermo Scientific) and Agilent Bioanalyser 2100 systems. Finally, the library was sequenced on an IlluminaHiSeq2500 platform, and 250 bp paired-end reads were generated.

### Isolation of cultivable bacteria

The homogenized larvae tissue was placed into 100 μL PBS solution, and 100 µL aliquots from samples were further serially diluted up to 10^−6^ and spread on Nutrient Agar (NA) (0.5% peptone, 0.3% beef extract, 1.5% agar, pH 6.8) (Difco, France) for the isolation of bacteria. Incubations were performed at 28 °C for 48 hours. Next, randomly selected single colonies were transferred to fresh NA plates. Selection of colonies was based on colony characteristics such as shape, colony size, colour, margin, elevation, opacity, and consistency.

### DNA extraction from cultured bacteria and sequencing

Randomly selected bacterial colonies were incubated in 5 mL Luria-Bertani broth (10 g/L Bacto Tryptone (Oxoid, Canada), 5 g/L Bacto-yeast extract (Oxoid, Canada), 5 g/L NaCl, pH 7.0) on a rotary shaker at 250 rpm at 28 °C overnight. Bacterial suspensions were centrifuged at 10000 rpm for 1 min and treated with proteinase K. Genomic DNA from bacterial isolates was extracted using a TIANamp Bacteria DNA Kit (Tiangen, China) following the manufacturer’s protocol. Extracted DNA was diluted 20-fold and used for polymerase chain reaction (PCR) on a Veriti thermocycler (Applied Biosystems, USA). Forward primer 27 F 5′-AGAGTTTGATCATGGCTCAG-3′ and reverse primer 1492 R 5′-TACGGCTACCTTGTTACGACTT-3′ were used for PCR amplification^40^. Amplifications were performed in a total volume of 50 µL containing 10 µL of PrimeSTAR HS (Premix) (Takara, Japan) containing an appropriate concentration of dNTPs (0.2 mM) and Taq polymerase (5 U), 1 µL (0.2 µM) of each primer, and 2 µL of diluted DNA. The PCR conditions included 5 min at 95 °C for the initial step followed by 35 cycles at 94 °C for 15 s (denaturation), 55 °C for 30 s (annealing), and 72 °C for 2 min (elongation), with a final extension at 72 °C for 10 min. PCR products were visualized on a 1.5% agarose gel. PCR products were triplicated and sent to the company for further purification and Sanger sequencing at Quintara Bio (China).

### Sequence analysis

Paired-end reads were generated based on their unique barcode by removal of barcode and primer sequences. Next, paired-end reads were merged using FLASH V1.2.7 (http://ccb.jhu.edu/software/FLASH)^41^ for merging paired-end reads for further generating raw tags. Raw tags were filtered under specific filtering conditions to obtain high-quality clean tags^42^ following QIME V1.7.0 (http://qiime.org/index.html) for the quality-control process. Furthermore, tags were compared with the “Gold” database (http://drive5.com/uchime/uchime_download.html) to remove chimeric sequences^43,44^ to obtain effective tags. High quality tags were clustered into operational taxonomic units (OTUs) at 97% similarity using UPARSE (v. 7.0.1001)^45^. Sequences for representative OTUs were classified using the Ribosomal Database Project classifier (RDP)^46^ in the GreenGene Database^47^. All raw sequences were submitted to NCBI under Bioproject PRJNA488360 and SRA number SRP072036.

### Phylogenetic tree analysis

Sequences obtained with the Sanger method were assembled using SeqMan (DNASTAR Lasergene 7). Sequences of approximately 1400 bp were compared with other 16S RNAs deposited in the nucleotide collection in GenBank using the BLASTN algorithm. Representative OTUs and sequences from the Sanger method were aligned with CLUSTALW. A phylogenetic tree was constructed based on the UPGMA algorithm following the Tajima-Nei model with 1000 bootstrap replicates in MEGA7.

### Lignocellulolytic assays

A total of 288 cultivable aerobic bacterial isolates were grown in different media to determine their enzymatic activity. We evaluated the presence of different pathways of lignocellulolytic activity that are likely involved in the degradation of cell-wall components such as lignin, cellulose, *β*-D-xylan, *β*-D-cellobiose, and *β*-D-glucans. To test the lignocellulolytic activity of each pathway, assays were performed in different media supplemented with specific substrates or with direct application onto bacterial colonies. Enzymatic activity was determined by colour change or appearance of halos.

Cellulose hydrolysis was determined using carboxymethylcellulose (CMC, Sigma), which was supplemented into CMC medium (0.94 g/L KH_2_PO_4_, 1.9 g/L K_2_HPO_4_, 1.6 g/L KCl, 1.43 g/L NaCl, 0.15 g/L NH_4_Cl, 0.037 g/L MgSO_4_·7H2O, 0.017 g/L CaCl_2_, 0.1 g/L yeast extract, 7.5 g/L CMC, and 15 g/L agar, pH 7.0). Individual bacterial isolates were grown on CMC medium as the sole carbon source for 96 hours. After bacterial incubation, bacterial colonies were washed with water. Then, agar plates were stained with 0.5% Congo red solution for 30 min until CMC became dye-bound^17^. Furthermore, plates were rinsed with 1 M NaCl for 5 min to fix the coloration and then washed with water to clearly observe halos^48^.

Ligninolytic activity was determined by employing MEA-Remazol Brilliant Blue R (MEA-RBBR) supplemented into agar medium^49,50^. Bacterial inoculates were incubated on solid media with MEA-RBBR (NA medium, 0.02% wt/vol MEA-RBBR, pH 7.0) at 28 °C for 15 days. The presence of a decolorized area around the colony indicated microbial ligninolytic activity^50^. For the lignin oxidation assay, bacterial isolates were cultured in NA medium at 28 °C for 16 hours until healthy colonies were visible. Next, to determine lignin oxidation, cool filtered 1 mM 2,2′-azino-bis 3-ethylbenzothiazoline-6-sulfonic acid (ABTS, Sigma) was poured over the plates. A substrate colour change to green indicated laccase activity^17^.

Glucanase, xylanase and cellobiase activities were determined using specific substrates, applying them directly on bacterial colonies^27,30^. Briefly, substrates 10 mM 4-nitrophenyl *β*-D-glucopyranoside (Sigma), 4-nitrophenyl *β*-D-xylopyranoside (Sigma), and 4-nitrophenyl *β-*D-cellobioside, for *β*-glucanase, *β*-xylanase, and *β*-cellobiase, respectively, were dissolved at 0.6% w/v in 50 mM ammonium acetate buffer, pH 5.0. A drop of these solutions was placed directly on the bacterial colonies, and the plates were incubated at room temperature for 8 hours. The catalytic activity of the microbial enzymes was estimated by the yellow coloration of the substrates, indicating hydrolysis to liberate the *p*-nitrophenol group (4NP).

### Analysis of lignin

Lignin content was determined based on analyses of acid soluble lignin (AISL) (Klason lignin) and insoluble lignin (ASL)^51^. Briefly, the lignin fractionated into acid insoluble and soluble lignin. The nearest 10 mg of stem, bark, cambium, phloem, and insect frass materials were ground in liquid nitrogen, weighed and dried at 300 °C for 3 hours. Then, 150 µL of 72% sulfuric acid was added and held at room temperature for 1 hour, and samples were vortexed every 10 min. Then, 4200 µL of milli-Q water was added to dilute the acid to a 4% concentration. Samples were autoclaved at 121 °C for 1 hour, and hydrolysates were allowed slowly cool down to room temperature. Samples were centrifuged at high rpm for 1 min, and the supernatant was removed into a new tube for determination of acid soluble lignin by UV-vis (www.thermofisher.com) spectroscopy at 205 nm. The acid soluble^52^ and insoluble^53^ lignin contents were calculated using the following equations:

$$AISL \% =\frac{A}{W}\times 100$$

where AISL% = amino acid insoluble lignin, A = weight of lignin and W = oven-dried weight of test specimen, mg.

$$ASL \% =\frac{{\rm{abs}}}{{\rm{Coef}}}\times \frac{{\rm{V}}\times 100}{{\rm{W}}}$$

where ASL% = amino acid soluble lignin, abs = absorbance of sample, V = volume of solution, Coef = known coefficient of plant (here the poplar coefficient = 18.21), and W = amount of starting material.

### Statistical analysis

StatView software packages were used to perform Fisher’s PLSD test following an ANOVA (SAS Institute Inc., Cary, NC, USA).

## Data Availability

Accession Codes: Raw sequence data have been deposited in the Sequence Read Archive of the National Center for Biotechnology Information (SRA, NCBI) under the Bioproject (PRJNA488360) and the SRA (SRP160089).
